# Effects of Commercial Exergames vs. Traditional Indoor Exercise on Mood in Older Adults: A Randomized Controlled Trial

**DOI:** 10.3390/healthcare14111450

**Published:** 2026-05-24

**Authors:** Yingying Zhu, Xuanjia Ren, Jinho Yim, Yunxue Guan

**Affiliations:** 1 Department of Smart Experience Design, Graduate School of Techno Design, Kookmin University, Seoul 02707, Republic of Korea; summerlion@kookmin.ac.kr (Y.Z.); guanyunxue@kookmin.ac.kr (Y.G.); 2 School of Art and Design, Beijing Institute of Fashion Technology, No. 2 East Yinghua Road, North End of Heping Street, Chaoyang District, Beijing 100029, China; renxj@bift.edu.cn

**Keywords:** older adults, commercial exergames, mood states, leisure sports, healthy aging

## Abstract

Background/Objectives: With the development of the silver economy, older adults have shown increasing interest in digital technologies, such as electronic fitness games (Exergames). This study explores the impact of commercial exergames on the emotional experience of older adults in order to provide novel ideas and applications for healthy aging. Methods: This was a prospective, single-center, unblinded, repeated-measures randomized controlled trial comparing an exergame intervention with traditional indoor exercise. This study included 30 older adults (aged 60–89 years) who were able to move independently. The intervention group performed exergame training using Ring Fit Adventure, whereas the comparison group performed traditional indoor exercise. The intervention lasted four weeks, with two sessions per week (eight sessions). Mood states were assessed using the Brunel Mood Scale, and data were analyzed using a linear mixed-effects model to examine group, time, and interaction effects. Results: Significant group × time interaction effects were observed for confusion, depression, fatigue, tension, and vigor (*p* < 0.05). No significant interaction effect was found for anger (*p* = 0.942). Conclusions: This study examined commercial exergames from the perspectives of emotional experience and mental health. Both commercial exergames and traditional indoor exercise were associated with improvements in immediate mood states. The exergame-based training approach was associated with lower levels of confusion, depression, and fatigue, as well as higher vigor scores. The results provide preliminary evidence regarding the role of digital exercise in mood regulation among older adults.

## 1. Introduction

Between 2025 and 2050, the global population aged 60 years and older is expected to increase from 1.2 billion to 2.1 billion. With increasing age, older adults face increasing health challenges, including declines in physical functioning [1], cognitive functioning, and mental health [2]. Due to changes in social roles, loneliness, heart disease, stroke, and other noncommunicable and chronic diseases, the prevalence of mental disorders among older adults is rising [3]. Effective prevention and intervention for mental health problems in later life can help older adults maintain their independence and improve their quality of life [4], as well as encouraging older adults to participate more actively in family and community activities and strengthen their social connections [5].

Physical activity is widely recognized as effective in promoting healthy aging. The World Health Organization [6] states that older adults should engage in diverse forms of physical activity, including aerobic exercise to improve cardiorespiratory fitness, muscle exercise to maintain muscle strength, balance exercise to improve physical stability, and flexibility exercise to maintain body flexibility [7]. Sustained participation in physical activities can effectively reduce the risk of falls among older adults [8], enhance their functional abilities [9], and promote mental plasticity [10,11]. At the same time, the rapid growth of the silver economy and the increasing acceptance of digital technology among older adults affords them new opportunities for digital health interventions [12,13,14].

Commercial exergames combine physical exercise with interactive gaming elements, integrating entertainment, real-time feedback, and guided movements and potentially increasing motivation and participation in physical activity [15]. It has been reported that exergames may influence multiple aspects of older adults’ health [16]. Pacheco et al. found that exergames can improve balance and mobility in older adults [17]. A study by the National Institute on Aging found that exergames provide a novel environment that stimulates the hippocampus (the region of the brain responsible for memory storage and spatial positioning) [18], thus improving working memory and supporting the cognitive health of older adults [19], and other studies have reported other potential benefits for mental health [20,21]. For example, Otero observed that participants showed significant improvements in general health, physical functioning, social functioning, and mental health after a videogame intervention [22]. Bráulio et al. supported the value of exercise with the Xbox Kinect for sleep quality and anxiety in older adults [23]. However, these outcomes were evaluated as part of broader health constructs, making it difficult to determine how commercial exergames influence specific emotional responses.

Mood represents an immediate and sensitive indicator of psychological changes during and after exercise [24]. It is possible to predict performance and assess mental health by analyzing participants’ mood states [3,25]. Positive emotional responses during or after exercise may support motivation and continued participation, whereas negative mood experiences may increase the likelihood of dropout. Positive effects of exergames on overall health outcomes have been reported [26,27,28]; however, few studies have examined their independent effects on specific mood dimensions or compared them with traditional exercise conditions. Therefore, the mechanisms by which commercial exergames influence the mood states of older adults remain unclear.

This paper sought to explore the psychological impact of commercial exergames on older adults, with a particular focus on their mood dimensions. This study conducts an intervention experiment to examine whether participation in a specific exergame improves the mood states of older adults.

Research questions were as follows:

Question 1 (Q1): Does the mood state of older adults change over the exercise sessions?

Question 2 (Q2): Compared with traditional indoor exercise, can commercial exergames more effectively improve the mood state of older adults?

This study explores the effects of a commercial exergame on the mood states of older adults, thus examining health benefits different from beneficial physical and cognitive outcomes. The findings are expected to provide insight into the integration of smart devices with fitness activities to support the mental health of older adults.

## 2. Materials and Methods

### 2.1. Study Design

**Figure 1:**
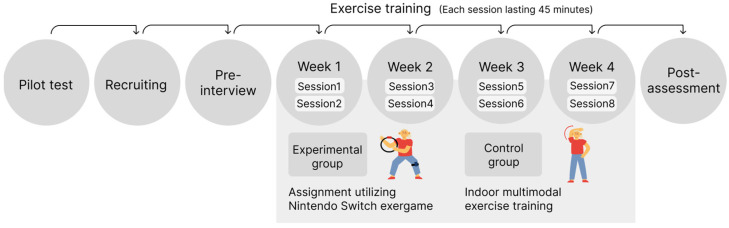
Study procedure and timeline of the randomized controlled trial.

This was a prospective, single-center, controlled trial with a repeated-measures design comparing commercial exergames with traditional indoor exercise. The overall study flow is illustrated in Figure 1. The experimental procedure consisted of three stages. First, a preliminary survey collected participants’ characteristics and their existing understanding of commercial exergames. Second, participants were randomly assigned to two groups. In an introductory session, the tasks were explained that participants would complete over the following month. Participants were asked to practice for 5 to 10 min and were allowed to pause or repeat the tasks as needed. Third, the intervention was implemented over four weeks, with two sessions per week (for a total of eight sessions). Each session lasted approximately 45 min.

Patients and the public were not involved in the design, conduct, reporting, or dissemination plans of this study. No changes were made to the trial protocol, eligibility criteria, outcomes, or analysis plan after the trial commenced. No interim analyses were planned, given the short intervention period and low-risk nature of the study.

### 2.2. Participants

Researchers recruited older adults from a public nursing home in southern China. Recruitment materials were sent to the director of the nursing home by email. After approval was obtained from the administrator, participants were recruited through on-site visits and face-to-face communication. The research objectives and procedures were explained to the candidates, after which they were assessed under the following inclusion criteria: (1) Aged 60 years or older; (2) no cognitive impairment (Mini-Mental State Examination score greater than or equal to 24); (3) normal hearing and vision or corrected to normal; and (4) no limb dysfunction or musculoskeletal problems that would restrict safe exercise participation, and able to move freely and independently. All participants volunteered to participate after being informed of the study procedures, and upon agreeing, provided written informed consent in File S1. Participants were instructed to maintain their usual daily routines and to avoid additional structured exercise programs during the study period.

### 2.3. Trial Setting

The sessions were conducted in an empty room adapted for exergame testing at the nursing home. Holding the sessions in a familiar environment helped reduce older adults’ anxiety toward the equipment and experimental procedures.

### 2.4. Intervention and Comparator

The study examined participants under two conditions: (1) the intervention group: Nintendo Switch game Ring Fit Adventure (Nintendo Co., Ltd., Kyoto, Japan); and (2) the comparison group: traditional indoor exercise.

The traditional indoor exercise practiced by the comparison group was based on the combined exercise model proposed in the Consensus of Chinese Experts on Sports Prescriptions [29] and was designed with four components: aerobic exercise, muscle exercise, balance exercise, and flexibility exercise. To ensure safety, exercise intensity was adjusted according to exercise guidelines for older adults [30]. For example, participants could use a chair for support when performing one-leg standing exercises. File S2 presents a sample set of traditional indoor exercises, which was designed to match the physical capabilities of older adults and prevent injuries from excessive exercise intensity.

Commercial exergames were represented in this study by Nintendo’s Ring Fit Adventure, an action role-playing game that combines adventure with physical exercise. It encourages players to defeat on-screen enemies by performing physical movements. The system includes four physical components: a ring-shaped Ring-Con, leg accessories, and two console handles (Joy-Con). Participants hold the Ring-Con in their hands, attach the controller sensor to their thighs, and perform exercises according to the instructions displayed on the screen. Each fitness movement in Ring Fit Adventure is associated with specific training keywords (aerobic, core, etc.), making the exercises easier to understand and select.

The exergame provided participants with an interactive, narrative-driven exercise environment. During the activity, participants received real-time feedback through visuals, music, and controller vibrations. The digital system incorporated gamified motivational features, such as virtual rewards and completion scores. In contrast, although the comparison group followed the same core exercise objectives, all gamification elements and immersive feedback were absent.

To ensure comparability between the intervention and comparison groups, this study selected 10 representative movements from the exercise modules of Ring Fit Adventure. First, these movements were of moderate intensity, were suitable for the physical capabilities of older adults, and were safe for repeated practice during the four-week training period. Second, the selected movements corresponded to the four exercise components used in traditional training. For example, the aerobic exercise module included “Side Step” and “Ring Raise Combo”, while the flexibility module featured “Warrior Pose”, “Overhead Arm Twist”, and “Chair Pose”. File S3 presents examples of the 10 exercises chosen from those included in Ring Fit Adventure. Both groups followed the same standardized training structure in each session, consisting of warm-up, training, and cool-down phases, with a total duration of 45 min. The sequence, duration, and intensity of movements were kept identical across sessions to ensure consistent intervention delivery.

### 2.5. Sample Size and Randomization

An a priori power analysis was conducted using G*Power 3.1 (Heinrich Heine University Düsseldorf, Düsseldorf, Germany) to determine the required sample size. The analysis model was specified as a repeated measures design. The significance level was set at 0.05 and the statistical power at 0.80. Drawing upon previous studies examining the effects of exercise interventions on mood states [23,31], a medium effect size (f = 0.25) was assumed. The analytical model included a between-subject factor (intervention group vs. comparison group) and a within-subject time factor (T1–T8) to test interactions between group and time. The calculation indicated that the minimum required sample size was approximately 28 participants (14 per group). Given the challenges of recruiting and retaining older adults, the target sample was set slightly above the estimated minimum. The repeated measures design across eight sessions allowed each participant to contribute multiple observations over time, increasing the amount of data available for analysis.

A random number sequence was generated using IBM SPSS Statistics 27.0 (IBM Corp., Armonk, NY, USA) with an allocation ratio of 1:1. Block randomization with variable block sizes of 4 and 6 was used to ensure unpredictability. The random sequence generation and participant allocation were conducted by an independent researcher who was not involved in recruitment, intervention, or outcome assessment. The allocation sequence was kept by this researcher and was not accessible to the study investigator or recruitment personnel before participant enrollment. The study investigator enrolled participants, and group assignment was performed according to the pre-generated random sequence to ensure blind allocation. Outcome assessments were conducted by trained research staff who were not involved in the randomization process. Due to the obvious differences in equipment used in the two intervention conditions, blinding of participants and intervention administrators was not feasible.

### 2.6. Outcome Measures

At the pre-interview stage, participants completed a demographic questionnaire regarding their basic information. They were also asked about their weekly exercise habits, such as the number of exercise days per week and average duration of exercise.

The mood state of the participants was evaluated using the Brunel Mood Scale (BRUMS). File S4 presents the specific contents of the scale. The scale contains five negative factors (anger, confusion, depression, fatigue, tension) and one positive factor (vigor) [32]. BRUMS is simplified from the Profile of Mood States, consisting of 24 items reduced from the original 65 items while maintaining the confidence and sensitivity of mood state assessment of the original scale [33]. The BRUMS [34] is widely used in research on physical activity. For example, it has been used to examine the mood states of university students during VR-based cycling [35] and to assess the motivational effects of listening to music before athletic competitions [36]. Table 1 presents the questionnaire items and basic definitions for each dimension of the BRUMS.

The BRUMS has been translated into multiple languages and is suitable for diverse cultural contexts. The version used in this study was the Chinese translation by Zhang [37]. Each subscale consists of 4 items, for a total of 24 questions. Participants rated their current feelings using prompts such as “How do you feel right now?” or “Please select the word on the list that best describes your mood.” Responses were scored on a 5-point Likert scale to indicate the intensity of their emotional experience after completing the task (0 = Not at all, 1 = A little, 2 = Moderately, 3 = Quite a bit, 4 = Extremely).

Data collection and intervention sessions were conducted by trained research staff following predefined procedures. Research staff received training on study protocols, participant guidance, and questionnaire administration before the study. Each session was supervised on a one-to-one basis to ensure safety. The BRUMS questionnaire was administered immediately after each session using the same procedure for both groups. Participants completed the questionnaire independently. All responses were checked for completeness after each session.

### 2.7. Statistical Methods

Research data were analyzed using IBM SPSS Statistics 27.0. Data analysis was performed in two steps. First, descriptive statistics were used to summarize participants’ baseline demographic characteristics, including age, gender, marital status, and education level, and pre-intervention BRUMS scores for the six mood dimensions. Continuous variables are presented as mean (SD), categorical variables as *n* (%). Between-group differences at baseline were examined using independent-samples tests. When the assumption of equal variances was violated, Welch’s test results were reported. Raw means and standard deviations were then calculated to provide an initial overview of observed mood changes across the eight sessions for the two groups. Second, the quantitative data were analyzed using linear mixed-effects models (LMMs). Group (intervention vs. comparison), time (T1–T8), and group × time interaction were included as fixed effects, and participants were treated as random effects. Estimated marginal means (EMMs) and their corresponding 95% confidence intervals (CIs) were derived from the LMMs to illustrate the statistically adjusted trajectories of mood scores. A significant interaction term (*p* < 0.05) would indicate statistically distinct trajectories of mood score change between the two groups. For significant group × time interaction effects, post hoc pairwise comparisons between the intervention and comparison groups at each session were conducted based on the EMMs. Bonferroni adjustment was applied to these post hoc comparisons to control for multiple testing, with the overall significance level set at α = 0.05. Adjusted *p*-values were reported for all post hoc comparisons. The fixed effects were reported as estimated regression coefficients (β) with their corresponding 95% CIs to indicate the direction and magnitude of the effects. To improve the interpretation of effect magnitude, standardized beta estimates (Std. β) were reported for the between-group effects of the six BRUMS mood dimensions.

All analyses were conducted according to the intention-to-treat principle and followed the prespecified study protocol. No subgroup or sensitivity analyses were planned or performed beyond the predefined between-group comparisons. All statistical tests were two-sided, with a significance level of α = 0.05. The assumptions for the valid application of LMMs were assessed prior to analysis, including the normality of residuals, homogeneity of variance, and independence of observations. The normality of the data was assessed using the Shapiro–Wilk test, which is appropriate for small sample sizes [38].

### 2.8. Ethical Considerations

This study followed the Consolidated Standards of Reporting Trials (CONSORT) 2025 statement and the CONSORT-EHEALTH guidelines to improve the transparency and reproducibility of digital health intervention research, which was shown in Files S5 and S6 [39,40].

This study was approved by the Ethics Committee of Zhejiang University (approval number: CMIC2025093). The study protocol was prospectively registered in the Chinese Clinical Trial Registry (registration number: ChiCTR2500111853) on 6 November 2025. During the recruitment stage, all participants were informed about the study topic, procedures, and their rights. To protect participants’ privacy and confidentiality, all collected data used in this study were deidentified. After the study, each participant received a small compensation in the form of daily necessities or a gift card (approximately US $5).

## 3. Results

### 3.1. Participant Flow and Recruitment

**Figure 2:**
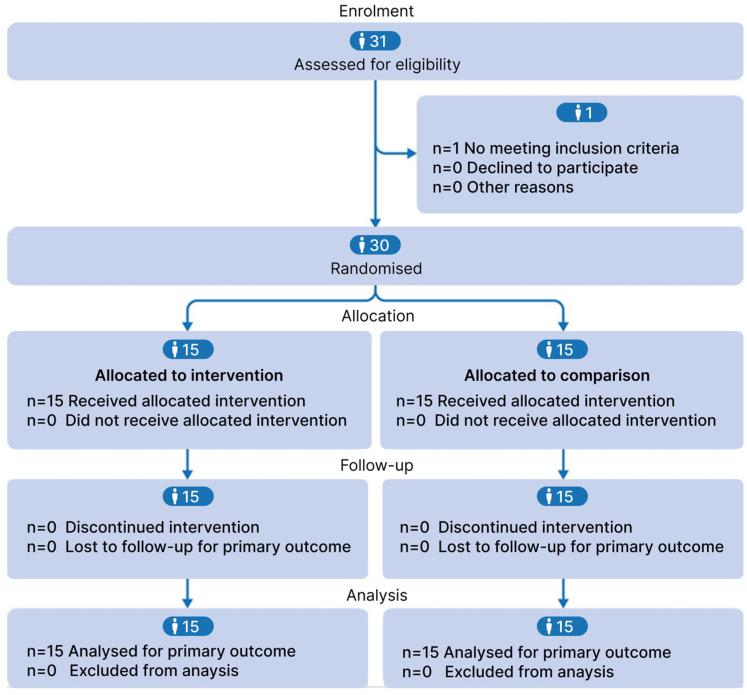
Consolidated Standards of Reporting Trials (CONSORT) 2025 flow diagram of participant recruitment, allocation, follow-up, and analysis.

The recruitment was completed between November 2025 and December 2025. A total of 31 individuals were screened, but one participant was excluded for not meeting the inclusion criteria. The remaining 30 eligible participants were randomly assigned to either the intervention group (*n* = 15) or the comparison group (*n* = 15). Figure 2 shows the enrollment and allocation flow chart of participants. All 30 participants completed all eight intervention sessions, and no participants dropped out during the study. Therefore, the session-level adherence rate was 100%. As there were no missing data in the final analytic sample, no imputation procedures were required. All randomly assigned participants were included in the primary analysis.

### 3.2. Intervention Delivery and Adherence

Attendance was recorded for each session to monitor adherence to the intervention protocol. Adverse events were monitored throughout the intervention period, in conjunction with which participants were asked to report any discomfort, pain, or dizziness during or after exercise. No adverse events were reported in either group.

### 3.3. Baseline Data

A total of 30 participants were included in the quantitative analysis, with 15 in each group. Baseline characteristics of participants are shown in Table 2. Participants were aged 60 to 89 years, with a median age of 69 years. The mean BMI was 23.22 ± 3.96 in the intervention group and 22.97 ± 5.55 in the comparison group. Participants reported engaging in physical activity 4.63 days per week, with a mean weekly exercise duration of 136 min. Regarding educational attainment, 13 participants (43.33%) had completed primary or junior high school, while 14 (46.67%) had attained a bachelor’s degree or higher. Most participants (25/30, 83.33%) reported no prior experience with exergames. Before the intervention period, participants completed the BRUMS once to assess their baseline mood state. Table 3 presents the baseline mood scores of the two groups. No significant between-group differences were observed in baseline BRUMS scores for anger, confusion, depression, fatigue, tension, or vigor, indicating that the groups were comparable in mood state before the intervention.

### 3.4. Outcomes

Table 4 presents the raw mean (SD) mood state scores for the intervention and comparison groups across the eight training sessions. Descriptive statistics showed that the intervention group had lower mean scores for Con, Dep, and Fat than the comparison group. In contrast, the intervention group showed higher scores for Ten and Vig. As higher scores represent poorer mood for all dimensions except vigor, these results reflect lower levels of confusion, depression, and fatigue but higher tension levels in the intervention group.

### 3.5. Linear Mixed-Effects Model Results

Table 5 summarizes the results for the LMMs regarding the effects of time, group, and their interaction on the six mood state dimensions. Parameter estimates (β) and corresponding 95% CIs were reported for the main effect of group to facilitate interpretation of between-group differences. Estimates for time and group × time interaction were not presented, as these effects involve multiple levels and are more appropriately interpreted using overall F tests rather than individual parameter estimates.

LMMs revealed significant main effects of time for all six mood dimensions: Ang (F(7, 96.85) = 2.58, *p* = 0.017), Con (F(7, 117.68) = 19.52, *p* < 0.001), Dep (F(7, 97.09) = 24.43, *p* < 0.001), Fat (F(7, 144.51) = 12.64, *p* < 0.001), Ten (F(7, 108.49) = 4.61, *p* < 0.001), and Vig (F(7, 109.34) = 31.13, *p* < 0.001). These results indicate that mood states changed significantly across the eight exercise sessions.

Significant main effects of group were observed for Con (F(1, 27.98) = 12.91, *p* = 0.002), Dep (F(1, 27.9) = 48.64, *p* < 0.001), Fat (F(1, 50.4) = 49.71, *p* < 0.001), Ten (F(1, 27.98) = 11.36, *p* < 0.001), and Vig (F(1, 27.99) = 24.76, *p* < 0.001). No significant group effect was found for Ang (F(1, 31.07) = 1.61, *p* = 0.213).

Linear mixed-effects models revealed significant group × time interaction effects for Con (F(7, 117.68) = 6.25, *p* < 0.001), Dep (F(7, 97.09) = 5.57, *p* < 0.001), Fat (F(7, 144.51) = 3.62, *p* < 0.001), Ten (F(7, 108.49) = 2.90, *p* = 0.008), and Vig (F(7, 109.34) = 13.12, *p* < 0.001), indicating differential changes in mood states over time between the intervention and comparison groups. No significant interaction effect was observed for Ang (F(7, 96.85) = 0.32, *p* = 0.942).

### 3.6. Group Effect Estimates

Parameter estimates for the group effect were coded as the value for the comparison group minus that for the intervention group. Thus, negative β values indicate higher scores in the intervention group, whereas positive values indicate higher scores in the comparison group.

Significant group effects were found for Con (β = 0.48, 95% CI [0.28, 0.69], Std. β = 1.28), Dep (β = 0.55, 95% CI [0.35, 0.75], Std. β = 1.36), Fat (β = 0.62, 95% CI [0.43, 0.81], Std. β = 1.66), Ten (β = −0.47, 95% CI [−0.67, −0.25], Std. β = −1.43), and Vig (β = −0.70, 95% CI [−0.92, −0.48], Std. β = −1.59). No significant group effect was found for Ang (β = −0.06, 95% CI [−0.29, 0.16], Std. β = −0.21). The relatively narrow 95% CIs observed for the key outcomes suggest that the estimated group differences were precise and robust.

### 3.7. Estimated Marginal Means

**Figure 3:**
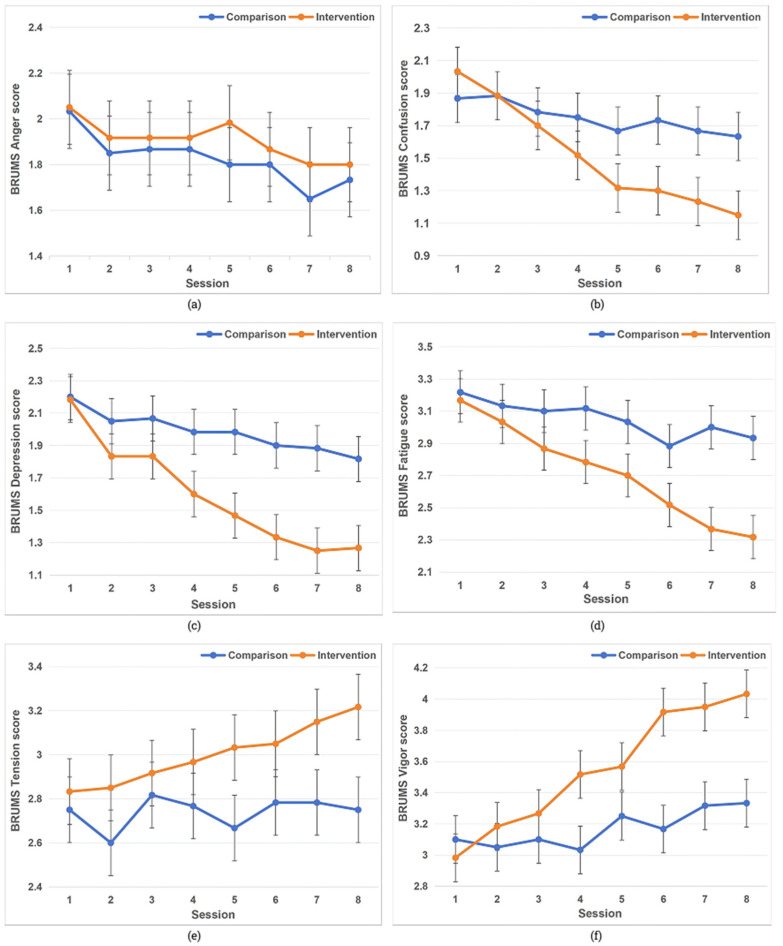
Estimated marginal means of the BRUMS scores for the intervention and comparison groups across eight sessions. Panels show (**a**) anger, (**b**) confusion, (**c**) depression, (**d**) fatigue, (**e**) tension, and (**f**) vigor. Error bars represent 95% CIs estimated from the LMMs. Higher vigor scores indicate better mood, whereas higher scores for the other dimensions indicate poorer mood states.

EMMs with 95% CIs estimated by the LMMs are presented in Figure 3. The figure provides a model-based visual overview of changes in BRUMS scores across the eight sessions. These visual patterns are further examined through Bonferroni-adjusted post hoc pairwise comparisons.

### 3.8. Post Hoc Pairwise Comparisons by Session

Post hoc pairwise comparisons based on estimated marginal means were conducted to clarify the specific sessions at which the two groups differed. The mean difference was calculated as the value for the comparison group minus that for the intervention group. Because Bonferroni-adjusted *p* values were reported, significance was interpreted at *p* < 0.05. The Bonferroni-adjusted post hoc pairwise comparison results for the six BRUMS dimensions are presented in File S7.

In summary, no significant differences between groups were observed for anger at any session. For confusion, significant differences emerged from session 4 through session 8, with the comparison group showing higher scores than the intervention group. For depression, significant differences were observed from session 2 through session 8. For fatigue, significant differences emerged from session 3 through session 8. The comparison group showed higher scores than the intervention group. For vigor, significant differences emerged from session 4 through session 8, with the intervention group showing higher scores than the comparison group. In contrast, for tension, the intervention group showed significantly higher scores than the comparison group at session 2 and sessions 5–8.

## 4. Discussion

### 4.1. Principal Results

This study examined the effects of commercial exergames and traditional indoor exercise on the mood states of older adults by addressing two research questions: (Q1) Did the mood states of older adults change over the course of the exercise intervention? And (Q2) Were commercial exergames more effective than traditional indoor exercise in improving the mood states of older adults? The results indicate an overall improvement in participants’ mood states during the exercise intervention. Both forms of exercise are associated with more favorable immediate mood states. Compared with traditional indoor exercise, exergame interventions were more effective in reducing confusion, depression, and fatigue while increasing vigor. Overall, as a form of exercise that integrates entertainment with physical activity, commercial exergames may have potential as an engaging approach to support short-term mood regulation in generally healthy older adults.

### 4.2. Interpretation of the Findings

Vigor refers to an individual’s subjective feeling of energy, reflecting a state of activity and vitality and representing a core dimension of positive mental health [41]. The intrinsic aspect of vigor reflects the independence and autonomy of the individual, whereas the external connection is related to social relations and social participation [42,43]. This study observed that participants’ vigor exhibited a positive trend as the exercise sessions progressed, a finding consistent with previous studies of improvements in subjective vitality through specialized training [44]. Anderiesen found that among patients with moderate to severe dementia, serious games may reduce negative emotions (e.g., sadness, anger, and fear) while increasing positive emotions (e.g., happiness and vitality) [45]. This pattern may be partly related to the enjoyable, multisensory, and immersive nature of commercial exergames, which provide users with greater challenges and enjoyment than traditional physical activities [46]. For example, one participant stated, “I am very interested in collecting gold coins. I can use gold coins to buy clothes and food, and I count how many coins I collect each time.” This suggests that the additional reward mechanisms provide motivation and a sense of accomplishment that enhance individuals’ mental energy [47]. These elements may influence mood regulation by transforming repetitive physical activity into goal-oriented and feedback-rich experiences.

Among older adults, depression often manifests as loss of interest and low mood and is associated with mental disorders and social isolation [48]. Confusion refers to feelings of helplessness and frustration, often accompanied by reduced attention and orientation. Fatigue is characterized by a persistent sense of physical exhaustion, reflected in indifference toward external stimuli and extreme tiredness [49]. In the present study, participants in the intervention group reported lower scores for confusion, depression, and fatigue compared with those in the comparison group. These findings are broadly consistent with those of earlier research. For instance, Swinnen et al. examined the effects of exergames on lower-limb function, cognitive performance, step reaction time, and depressive symptoms in patients with major neurocognitive disorder, finding that the intervention group showed significantly greater reductions in depression scores than the control group [50]. Older adults may choose exergames as a form of exercise because they foster a sense of social connection and participation [51], reflecting the fact that players often enjoy cooperating or competing with their family members or peers, which may reduce feelings of loneliness and strengthen their sense of social belonging [52]. Some studies also suggest that exergames can improve cognitive abilities in older adults, indirectly alleviating fatigue and depressive symptoms [53]. However, individual differences and the level of intervention should be taken into account, as excessive exercise may intensify negative effects and constitute behavioral addiction [54].

This study observed higher tension scores in the intervention group at certain time points than in the comparison group. Within the BRUMS framework, tension is typically classified as a negative mood dimension associated with anxiety or nervousness [55]. This finding suggests that emotional responses in exergame-based exercise may present a more complex picture [56]. One possibility is that participants might have experienced increased cognitive demands or task-related challenges when interacting with a novel technology [57,58]. This exercise format involves the use of controllers and dynamic visual changes, which might have been unfamiliar to some of the older adults, thereby causing them some degree of uncertainty reflected in the higher tension scores [59]. Previous studies have reported mixed findings regarding changes in tension or anxiety in exercise or gaming contexts. For example, Xu et al. found no significant differences in anxiety scores in immersive virtual reality game conditions [60]. In contrast, Viana et al. suggested that regular exercise can alleviate anxiety symptoms, with improvements associated with adherence to the intervention [61]. It should be noted that the tension discussed in this study differs from clinical pathological anxiety [62]. As BRUMS was administered immediately after each session, the tension scores should not be taken as evidence of sustained anxiety. Participants in this study were generally healthy older adults. This study aimed to examine the potential role of mood regulation in improving quality of life rather than treating clinical anxiety disorders [63,64].

According to the theoretical framework proposed by Man [65], anger can be categorized into state anger [66] and trait anger [67]. This study focused on state anger, which reflects participants’ immediate emotional responses following exercise. The interaction between time and group for anger was not significant. The intervention did not alter the trajectory of anger over time or produce stable differences between groups at any single time point. Descriptive results suggest a gradual decrease in average anger scores across sessions, consistent with earlier findings. Exercise may reduce the induction of anger, but it does not change individuals’ responses to triggering events [68].

### 4.3. Limitations

This study has several limitations. First, although the sample size met the minimum requirement estimated by a priori power analysis, the final sample size remained relatively small. This may have increased the risk of Type I and Type II errors and potentially led to overestimation of the intervention effects. The findings should be interpreted as preliminary. Second, the instrument used in this study (BRUMS) primarily measures participants’ immediate mood states in specific situations. In other words, the results mainly reflect the short-term emotional regulation effects of exercises, while their impact on long-term mental health outcomes (e.g., persistent depression or anxiety symptoms) remains unverified. No follow-up assessment was conducted after the intervention, so it is unclear whether the observed changes were maintained over time. Third, this study did not include measures of exercise intensity, such as heart rate, Rating of Perceived Exertion, or metabolic equivalent values. Although the two interventions followed similar exercise structures, the actual physiological load may have differed between groups. Future studies should monitor exercise intensity better to distinguish the effects of gamification from physiological exercise load. Fourth, participants were recruited from a single study site within a limited geographic region, which may restrict the generalizability of the findings. Future multisite studies are needed to improve external validity. Fifth, blinding of participants and intervention providers was not feasible due to the distinct characteristics of the two exercise conditions. Mood states were assessed using the self-reported Brunel Mood Scale. Participants’ awareness of the intervention type may have influenced their responses through expectation bias or the Hawthorne effect. Therefore, the observed changes in mood states should be interpreted with caution. Finally, the study included generally healthy older adults who were able to exercise independently. The results thus may not generalize to individuals with significant physical or cognitive limitations. Future studies should recruit a broader range of participants and incorporate supportive measures to facilitate participation among older adults with varying physical, cognitive, and digital abilities.

## 5. Conclusions

This study provides new insights into the possible advantages of employing commercial exergames in short-term mood regulation among older adults. Both commercial exergames and traditional indoor exercise were associated with improvements in immediate mood states. Compared with traditional indoor exercise, exergame-based training showed lower levels of confusion, depression, and fatigue, along with higher vigor scores. The value of exergames may extend beyond physical activity to include emotional experience during exercise. From a theoretical perspective, the results contribute to understanding the short-term mood benefits of commercial exergames in older adults. From a practical perspective, commercial exergames may represent an engaging form of exercise for older adults. The findings may also hold design implications for developers creating health-oriented games for older adults. As an exploratory study, this work may help inform future research on the potential role of commercial exergames in supporting healthy aging. Future studies with larger samples, longer intervention periods, and follow-up assessments are needed to extend the present findings.

## Figures and Tables

**Table 1:** Dimensions and item composition of the Brunel Mood Scale.

Dimension	Item	Definition
Tension	9, 16, 17, 18	The state of musculoskeletal rigidity and psychological concern.
Anger	3, 8, 10, 11	Reflects a state of hostility directed toward others. This mood dimension is linked to antipathy.
Depression	1, 2, 20, 22	An emotional state characterized by discouragement, sadness, and general unhappiness.
Confusion	4, 5, 7, 19	A state of mental uncertainty and emotional instability.
Fatigue	6, 21, 23, 24	Defined as a tired condition resulting from an energy deficit.
Vigor	12, 13, 14, 15	A state of high energy and physical vitality.

**Table 2:** Baseline characteristics of participants in the intervention and comparison groups (N = 30).

Characteristic	Total (N = 30)	Intervention Group (*n* = 15)	Comparison Group (*n* = 15)
Age (Year) (Mean ± SD)	69.20 ± 7.64	72.13 ± 8.34	66.27 ± 5.74
Gender (*n* (%))			
1. Female	16 (53.33)	8 (53.33)	8 (53.33)
2. Male	14 (46.67)	7 (46.67)	7 (46.67)
BMI * (kg/m^2^) (Mean ± SD)	23.09 ± 4.74	23.22 ± 3.96	22.97 ± 5.55
Weekly exercise frequency (Day) (Mean ± SD)	4.63 ± 2.37	3.67 ± 2.44	5.60 ± 1.92
Total weekly physical activity (Minutes) (Mean ± SD)	136.00 ± 96.23	127.33 ± 89.00	144.67 ± 105.35
Marital status (*n* (%))			
1. Married	20 (66.67)	8 (53.33)	12 (80)
2. Single	2 (6.67)	1 (6.67)	1 (6.67)
3. Divorced	1 (3.33)	1 (6.67)	0 (0)
4. Widowed	7 (23.33)	5 (33.33)	2 (13.34)
Education level (*n* (%))			
1. Junior High School 2. Senior High School	13 (43.33)	8 (53.33)	5 (33.33)
	3 (10)	1 (6.67)	2 (13.33)
3. Undergraduate	14 (46.67)	6 (40)	8 (53.33)
Exergame use (*n* (%))			
Yes	5 (16.67)	3 (20)	2 (13.33)
No	25 (83.33)	12 (80)	13 (86.67)

* BMI: Body Mass Index. SD: standard deviations. Percentages are calculated within each column (Total N = 30; Intervention *n* = 15; Comparison *n* = 15). Percentages may not sum to 100 due to rounding.

**Table 3:** Baseline BRUMS scores of participants in the intervention and comparison groups.

Baseline BRUMS Scores (Mean ± SD)	Intervention Group	Comparison Group	*p* Value
Ang *	2.13 (0.64)	2.20 (0.77)	0.799
Con	2.07 (0.59)	1.93 (0.26)	0.432
Dep	1.93 (0.70)	2.20 (0.77)	0.332
Fat	2.40 (0.51)	2.67 (0.49)	0.153
Ten	2.07 (0.70)	2.00 (0.38)	0.749
Vig	2.80 (0.56)	3.07 (0.46)	0.165

* Ang: anger; Con: confusion; Dep: depression; Fat: fatigue; Ten: tension; Vig: vigor.

**Table 4:** Descriptive statistics for BRUMS scores in the intervention and comparison groups.

	Ang *	Con	Dep	Fat	Ten	Vig
Scores (Mean ± SD)						
Intervention	1.91 ± 0.31	1.52 ± 0.22	1.59 ± 0.27	2.72 ± 0.39	3.00 ± 0.30	3.55 ± 0.47
Comparison	1.83 ± 0.31	1.75 ± 0.14	1.98 ± 0.25	3.05 ± 0.27	2.74 ± 0.29	3.17 ± 0.31

* Ang: anger; Con: confusion; Dep: depression; Fat: fatigue; Ten: tension; Vig: vigor; SD: standard deviations.

**Table 5:** Linear mixed-effects model results for BRUMS mood state scores across the eight exercise sessions.

Fixed Effect	Time		Group		Time × Group	
	F Test (df1, df2)	*p* Value	F Test (df1, df2)	*p* Value	F Test (df1, df2)	*p* Value
Ang *	2.58 (7, 96.85)	0.017	1.61 (1, 31.07)	0.213	0.32 (7, 96.85)	0.942
Con	19.52 (7, 117.68)	<0.001	12.91 (1, 27.98)	0.002	6.25 (7, 117.68)	<0.001
Dep	24.43 (7, 97.09)	<0.001	48.64 (1, 27.90)	<0.001	5.57 (7, 97.09)	<0.001
Fat	12.64 (7, 144.51)	<0.001	49.71 (1, 50.4)	<0.001	3.62 (7, 144.51)	<0.001
Ten	4.61 (7, 108.49)	<0.001	11.36 (1, 27.98)	<0.001	2.90 (7, 108.49)	0.008
Vig	31.13 (7, 109.34)	<0.001	24.76 (1, 27.99)	<0.001	13.12 (7, 109.34)	<0.001

* Ang: anger; Con: confusion; Dep: depression; Fat: fatigue; Ten: tension; Vig: vigor.

## Data Availability

Deidentified data supporting the findings of this study are available from the corresponding author upon reasonable request, subject to ethical approval and compliance with the terms of participant consent. The data are not publicly available due to privacy and ethical restrictions related to human participant data.

## Supplementary Materials

The following supporting information can be downloaded at: https://www.mdpi.com/article/10.3390/healthcare14111450/s1, File S1: Informed consent form for older adult participants; File S2: Description of exercise components included in the traditional indoor exercise program; File S3: Description of exercise components in the Ring Fit Adventure exergame intervention; File S4: The Brunel Mood Scale (BRUMS) questionnaire used to assess mood states; File S5: CONSORT checklist; File S6: CONSORT-eHEALTH checklist (V 1.6.1); File S7: Post hoc pairwise comparisons.

## Abbreviations

The following abbreviations are used in this manuscript:

BRUMS	The Brunel Mood Scale
CONSORT	Consolidated Standards of Reporting Trials
CONSORT-eHEALTH	Consolidated Standards of Reporting Trials of Electronic and Mobile Health Applications and Online Telehealth
EMM	Estimated Marginal Mean
LMM	Linear Mixed Model
