# Supplementary material for: Effects of Commercial Exergames vs. Traditional Indoor Exercise on Mood in Older Adults: A Randomized Controlled Trial
Source: Healthcare (Basel). 2026 May 24;14(11):1450. doi: 10.3390/healthcare14111450 (PMC13257215; doi:10.3390/healthcare14111450)
Supplement: Supplementary file 1 [file healthcare-14-01450-s001.zip › File S1.pdf]

OLDER ADULTS INTERVIEW CONSENT FORM

Study Title: Effects of Commercial Exergames vs Traditional Indoor Exercise on Mood in Older Adults: A Randomized Controlled Trial

Understanding of my rights in research

Please circle YES or NO

I read the letter about this study.	**YES**	**NO**
I decided that I want to be in this study.	**YES**	**NO**
I know that I do not need to be in this study if I do not want to.	**YES**	**NO**
I will participate in this study. Each of the seesions will last 40-50 minutes.	**YES**	**NO**
I can stop my participation when I want.	**YES**	**NO**
It is okay for the researchers to use my answers when they tell people about their research.	**YES**	**NO**
I know that the researchers will not tell anybody my name.	**YES**	**NO**
It is okay for the researchers to ask me again if I want to continue participating in the study.	**YES**	**NO**

Participants:

I voluntarily consent to participate in the **subjective questionnaire** of the study "**Effects of Commercial Exergames vs Traditional Indoor Exercise on Mood in Older Adults: A Randomized Controlled Trial**". I understand that refusal to participate will have no effect on the services I receive at the Centre. I understand that I will receive a signed copy of this form.

___________________	____________________	___________________
Participant's Name (Print)	Participant's Signature	Date

By marking my initials here, _______, I agree to allow the researchers to contact me at a later time if they would like me to clarify any information.

Person obtaining consent:

I have discussed this study in detail with the participant. I believe the participant understands what is involved in this study.

_______________________	______________________	________________
Researcher's Name (Print)	Researcher's Signature	Date

If you have any questions regarding the ethical conduct of this study, you may contact College of Media and International Culture, Zhejiang UniversityWest District of Zijingang Campus866 Yuhangtang RoadHangzhou, Zhejiang, 310058 P.R. China. Tel.: +86-571-87075132, Email: 0922a42@zju.edu.cn
