# Supplementary material for: Effects of Commercial Exergames vs. Traditional Indoor Exercise on Mood in Older Adults: A Randomized Controlled Trial
Source: Healthcare (Basel). 2026 May 24;14(11):1450. doi: 10.3390/healthcare14111450 (PMC13257215; doi:10.3390/healthcare14111450)
Supplement: Supplementary file 1 [file healthcare-14-01450-s001.zip › File S2.pdf]

Supplementary Materials File S2: The exercise elements of traditional indoor sports

Program component	Duration	Action Style	Action Description	Categories
1. Warm up	5min	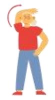	Raise arms behind the head	**Flexibility exercise**
		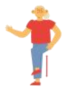	Alternating high knees	**Muscle exercise**
2. Exercise training	5min	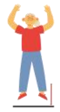	Jumping Jacks	**Aerobic exercise**
	20min	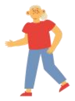	Brisk walking	**Aerobic exercise**
	3min	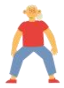	Bodyweight squat	**Muscle exercise**
	2min	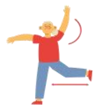	Single-leg stance	**Balance exercise**
	5min	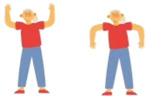	Swinging arms up and down	**Muscle exercise**
3. Cool down	5min	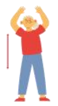	Yawning while stretching	**Flexibility exercise**
		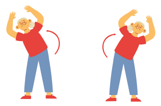	Lower back on the right and left side	**Flexibility exercise**
		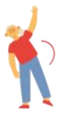	Raised hand extended to the side	**Balance exercise**

Action style image source from: Created by the authors
