# Supplementary material for: Effects of Commercial Exergames vs. Traditional Indoor Exercise on Mood in Older Adults: A Randomized Controlled Trial
Source: Healthcare (Basel). 2026 May 24;14(11):1450. doi: 10.3390/healthcare14111450 (PMC13257215; doi:10.3390/healthcare14111450)
Supplement: Supplementary file 1 [file healthcare-14-01450-s001.zip › File S3.pdf]

Supplementary Materials File S3: The exercise elements of Ring Fit Adventure

Program component	Duration	Action Name	Action Style	Action Description	Game visuals presentation	Categories
1. Warm up	5min	Overhead Arm Twist	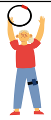	Raise both arms straight up, then twist your wrists.	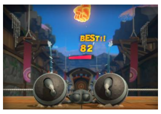	**Flexibility exercise**
		Standing Twist	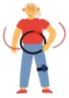	Twist your torso from side to side, with wide movements.	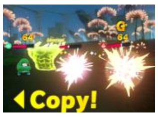	**Balance exercise**
2. Exercise training	10min	Side Step	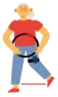	While stepping left and right, move the Ring-Conup and down.	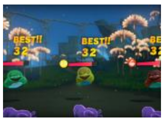	**Aerobic exercise**
	5min	Squat	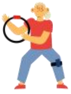	Drop your hips and plant your feet.	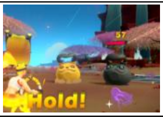	**Aerobic exercise**
	7min	Ring Raise Combo	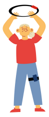	Move the Ring-Con up and down, matching the rhythm.	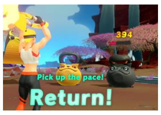	**Aerobic exercise**
	3min	Chair Pose	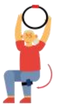	Drop your hips, then slowly move the Ring-Con up and down.	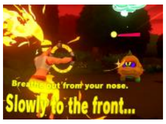	**Flexibility exercise**
	5min	Shoulder Press	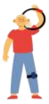	Hold the Ring-Con on one shoulder and squeeze it.	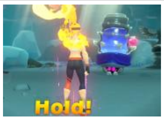	**Muscle exercise**
	5min	Knee-Lift Combo	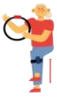	A combination exercise where you lift both your knees and the Ring-Con.	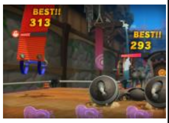	**Muscle exercise**
3. Cool down	5min	Warrior Pose	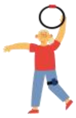	Place one leg far forward, spread your arms wide, then slowly twist both arms.	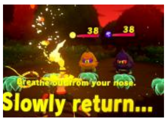	**Flexibility exercise**
		Overhead Side Bend	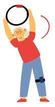	Arms Hold the Ring-Con overhead and lean your torsoside to side.	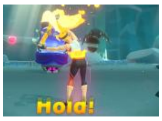	**Balance exercise**

Action style image source from: Created by the authors

Game visuals presentation image source from：

https://www.nintendo.com/hk/switch/ringadventure/list/index.html
