# Supplementary material for: Effects of Commercial Exergames vs. Traditional Indoor Exercise on Mood in Older Adults: A Randomized Controlled Trial
Source: Healthcare (Basel). 2026 May 24;14(11):1450. doi: 10.3390/healthcare14111450 (PMC13257215; doi:10.3390/healthcare14111450)
Supplement: Supplementary file 1 [file healthcare-14-01450-s001.zip › File S4.pdf]

Supplementary Materials File S4: The Brunel Mood Scale Questionnaire

Below is a list of words that describe feelings people have. Please read each one carefully and then circle the answer that best describes **HOW YOU FEEL RIGHT NOW**. Make sure you respond to every word.

| | Item | Hypothesised | Not at all | A little | Moderately | Quite a bit | Extremely |
|---|---|---|---|---|---|---|---|
| | | Factor | 0 | 1 | 2 | 3 | 4 |
| 1 | Depressed | Dep | | | | | |
| 2 | Downhearted | Dep | | | | | |
| 3 | Annoyed | Ang | | | | | |
| 4 | Mixed Up | Con | | | | | |
| 5 | Confused | Con | | | | | |
| 6 | Worn Out | Fat | | | | | |
| 7 | Muddled | Con | | | | | |
| 8 | Angry | Ang | | | | | |
| 9 | Nervous | Ten | | | | | |
| 10 | Bad tempered | Ang | | | | | |
| 11 | Bitter | Ang | | | | | |
| 12 | Energetic | Vig | | | | | |
| 13 | Active | Vig | | | | | |
| 14 | Lively | Vig | | | | | |
| 15 | Alert | Vig | | | | | |
| 16 | Anxious | Ten | | | | | |
| 17 | Worried | Ten | | | | | |
| 18 | Panicky | Ten | | | | | |
| 19 | Uncertain | Con | | | | | |
| 20 | Miserable | Dep | | | | | |
| 21 | Exhausted | Fat | | | | | |
| 22 | Unhappy | Dep | | | | | |
| 23 | Sleepy | Fat | | | | | |
| 24 | Tired | Fat | | | | | |

Note: Ang = anger, Con = confusion, Dep = depression, Fat = fatigue, Ten = tension, Vig = vigour.
