# Supplementary material for: Effects of Commercial Exergames vs. Traditional Indoor Exercise on Mood in Older Adults: A Randomized Controlled Trial
Source: Healthcare (Basel). 2026 May 24;14(11):1450. doi: 10.3390/healthcare14111450 (PMC13257215; doi:10.3390/healthcare14111450)
Supplement: Supplementary file 1 [file healthcare-14-01450-s001.zip › File S7.pdf]

Supplementary Materials File S7: Post-hoc Pairwise Comparisons

Mean difference was calculated as the comparison group minus the intervention group. Positive values indicate higher scores in the comparison group, whereas negative values indicate higher scores in the intervention group. SE = standard error; CI = confidence interval. Bonferroni adjustment was applied for multiple comparisons. For anger, confusion, depression, fatigue, and tension, higher scores indicate poorer mood states; for vigor, higher scores indicate better mood state.

1.Ang

Table S1: Bonferroni-adjusted post-hoc pairwise comparisons based on estimated marginal means for anger scores across eight sessions.

Session	Mean Difference	SE	95% CI	Bonferroni-adjusted p
1	-0.017	0.116	[-0.246, 0.213]	0.886
2	-0.067	0.116	[-0.296, 0.163]	0.567
3	-0.050	0.116	[-0.279, 0.179]	0.667
4	-0.050	0.116	[-0.279, 0.179]	0.667
5	-0.183	0.116	[-0.413, 0.046]	0.116
6	-0.067	0.116	[-0.296, 0.163]	0.567
7	-0.150	0.116	[-0.379, 0.079]	0.198
8	-0.067	0.116	[-0.296, 0.163]	0.567

For anger, no significant between-group differences were observed at any session after Bonferroni adjustment.

2. Con

Table S2: Bonferroni-adjusted post-hoc pairwise comparisons based on estimated marginal means for confusion scores across eight sessions.

Session	Mean Difference	SE	95% CI	Bonferroni-adjusted p
1	-0.167	0.106	[-0.377, 0.044]	0.119
2	0.000	0.106	[-0.210, 0.210]	1.000
3	0.083	0.106	[-0.127, 0.294]	0.434
4	0.233	0.106	[0.023, 0.444]	0.030
5	0.350	0.106	[0.140, 0.560]	0.001
6	0.433	0.106	[0.223, 0.644]	< 0.001
7	0.433	0.106	[0.223, 0.644]	< 0.001
8	0.483	0.106	[0.273, 0.694]	< 0.001

For confusion, Bonferroni-adjusted pairwise comparisons showed no significant between-group differences during Sessions 1 - 3. From Session 4 onward, the comparison group had significantly higher confusion scores than the intervention group, with mean differences increasing from 0.233 at Session 4 to 0.483 at Session 8, all $p \leq 0.030$.

3. Dep

Table S3: Bonferroni-adjusted post-hoc pairwise comparisons based on estimated marginal means for depression scores across eight sessions.

Session	Mean Difference	SE	95% CI	Bonferroni-adjusted p
1	0.017	0.100	[-0.181, 0.214]	0.868
2	0.217	0.100	[0.019, 0.414]	0.032
3	0.233	0.100	[0.036, 0.431]	0.021
4	0.383	0.100	[0.186, 0.581]	< 0.001
5	0.517	0.100	[0.319, 0.714]	< 0.001
6	0.567	0.100	[0.369, 0.764]	< 0.001
7	0.633	0.100	[0.436, 0.831]	< 0.001
8	0.550	0.100	[0.352, 0.748]	< 0.001

For depression, Bonferroni-adjusted pairwise comparisons showed no significant between-group difference at Session 1, mean difference = 0.017, SE = 0.100, 95% CI [-0.181, 0.214], p = 0.868. From Session 2 to Session 8, the comparison group had significantly higher depression scores than the intervention group, with mean differences ranging from 0.217 to 0.633, SE = 0.100, and Bonferroni-adjusted p values ranging from 0.032 to < 0.001.

4. Fat

Table S4: Bonferroni-adjusted post-hoc pairwise comparisons based on estimated marginal means for fatigue scores across eight sessions.

Session	Mean Difference	SE	95% CI	Bonferroni-adjusted p
1	0.050	0.096	[-0.139, 0.239]	0.603
2	0.100	0.096	[-0.089, 0.289]	0.299
3	0.233	0.096	[0.044, 0.423]	0.016
4	0.333	0.096	[0.144, 0.523]	0.001
5	0.333	0.096	[0.144, 0.523]	0.001
6	0.367	0.096	[0.177, 0.556]	< 0.001
7	0.633	0.096	[0.444, 0.823]	< 0.001
8	0.617	0.096	[0.427, 0.806]	< 0.001

For fatigue, Bonferroni-adjusted pairwise comparisons showed no significant between-group differences during Sessions 1 and 2. From Session 3 to Session 8, the comparison group had significantly higher fatigue scores than the intervention group. The mean differences ranged from 0.233 at Session 3 to 0.633 at Session 7, with all significant comparisons remaining significant after Bonferroni adjustment, p ≤ 0.016.

5. Ten

Table S5: Bonferroni-adjusted post-hoc pairwise comparisons based on estimated marginal means for tension scores across eight sessions.

Session	Mean Difference	SE	95% CI	Bonferroni-adjusted p
1	-0.083	0.106	[-0.294, 0.128]	0.435
2	-0.250	0.106	[-0.461, -0.039]	0.021
3	-0.100	0.106	[-0.311, 0.111]	0.349
4	-0.200	0.106	[-0.411, 0.011]	0.063
5	-0.367	0.106	[-0.578, -0.156]	0.001
6	-0.267	0.106	[-0.478, -0.056]	0.014
7	-0.367	0.106	[-0.578, -0.156]	0.001

8	-0.467	0.106	[-0.678, -0.256]	< 0.001

For tension, Bonferroni-adjusted pairwise comparisons showed no significant between-group differences at Sessions 1, 3, or 4. However, the intervention group showed significantly higher tension scores than the comparison group at Session 2 and Sessions 5 - 8. The mean differences ranged from -0.250 to -0.467, with all significant comparisons remaining significant after Bonferroni adjustment, $p \leq 0.021$.

6. Vig

Table S6: Bonferroni-adjusted post-hoc pairwise comparisons based on estimated marginal means for vigor scores across eight sessions.

Session	Mean Difference	SE	95% CI	Bonferroni-adjusted p
1	0.117	0.109	[-0.099, 0.332]	0.286
2	-0.133	0.109	[-0.349, 0.082]	0.223
3	-0.167	0.109	[-0.382, 0.049]	0.129
4	-0.483	0.109	[-0.699, -0.268]	< 0.001
5	-0.317	0.109	[-0.532, -0.101]	0.004
6	-0.750	0.109	[-0.966, -0.534]	< 0.001
7	-0.633	0.109	[-0.849, -0.418]	< 0.001
8	-0.700	0.109	[-0.916, -0.484]	< 0.001

For vigor, Bonferroni-adjusted pairwise comparisons showed no significant between-group differences during Sessions 1 - 3. From Session 4 to Session 8, the intervention group had significantly higher vigor scores than the comparison group. The mean differences ranged from -0.317 at Session 5 to -0.750 at Session 6, with all significant comparisons remaining significant after Bonferroni adjustment, $p \leq 0.004$.

Bonferroni-adjusted pairwise comparisons further clarified the specific sessions at which the groups differed. For confusion, significant between-group differences emerged from Session 4 and remained significant through Session 8. For depression, significant differences were observed from Session 2 through Session 8. For fatigue, significant differences emerged from Session 3 and remained significant through Session 8. In these three negative mood dimensions, the comparison group showed significantly higher scores than the intervention group. For vigor, significant differences emerged from Session 4 through Session 8, with the intervention group showing significantly higher scores than the comparison group. In contrast, for tension, the intervention group showed significantly higher scores than the comparison group at Session 2 and Sessions 5 - 8.
